# Electron Capture-Induced Charge Reduction Benefits the Recording of Ultralong Transients in Orbitrap-Based Individual-Ion Mass Spectrometry

**DOI:** 10.1021/acs.analchem.5c01000

**Published:** 2025-05-29

**Authors:** Manuel D. Peris-Díaz, Arjan Barendregt, Tobias P. Wörner, Kyle L. Fort, Alexander A. Makarov, Evolène Deslignière, Albert J. R. Heck

**Affiliations:** † Biomolecular Mass Spectrometry and Proteomics, Bijvoet Center for Biomolecular Research and Utrecht Institute for Pharmaceutical Sciences, Utrecht University, Utrecht 3584 CH, the Netherlands; ‡ Department of Chemical Biology, Faculty of Biotechnology, University of Wrocław, Wrocław 50-383, Poland; § Thermo Fisher Scientific GmbH, Hanna-Kunath-Straße 11, Bremen 28199, Germany

## Abstract

Recently, the use of ultralong transients has enabled exceptional resolution and sensitivity in Orbitrap-based charge detection mass spectrometry (CDMS). Nevertheless, measuring small analytes carrying a few charges remains a challenge. Prolonged trapping should, in theory, allow for the detection of lower charged ions (<10+) due to enhanced signal-to-noise (*S*/*N*) ratios. However, in practice, due to ion decay through frequency drifts, or collision-induced fragmentations, low *m*/*z* ions deviate from the ideal coherent trajectories in the Orbitrap. Here, by incorporating electron capture charge reduction (ECCR) in the gas phase prior to CDMS, we show that charge reduction significantly improves the stability of ion trajectories when ions are trapped for long periods in the Orbitrap analyzer. Using proteins with molecular weights ranging from 12 to 900 kDa, we demonstrate that ECCR-CDMS enhances ion survival by up to 60-fold, even enabling the detection of doubly charged individual ions from cytochrome c that typically elude conventional Orbitrap-based CDMS.

## Graphical abstract

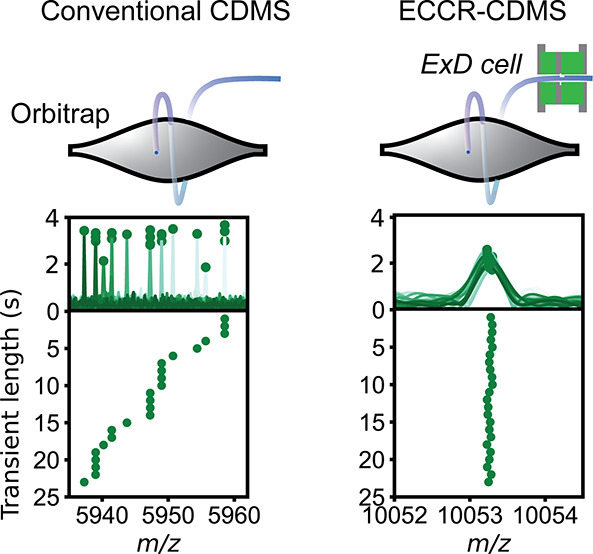

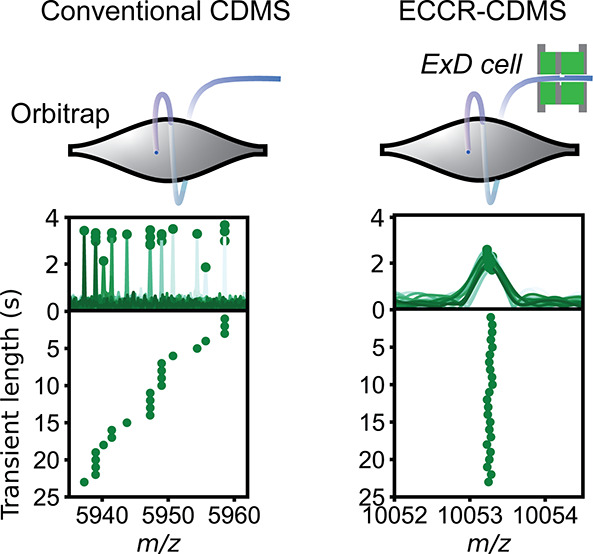

## Introduction

Charge detection mass spectrometry (CDMS) has emerged as a distinctive approach to characterize heterogeneous, high-mass assemblies such as viruses, vaccines, and heavily glycosylated therapeutic proteins.^1,2^ These large and often polydisperse samples typically display unresolvable mass spectra in conventional ensemble MS, hampering charge assignment and thus mass determination. CDMS instead detects individual ions, simultaneously measuring the charge (which in an Orbitrap mass analyzer scales directly with the ion’s intensity, albeit only when the ion survives the entire transient) and *m*/*z* of each particle, thereby avoiding the need for a resolved charge state distribution. Charge determination of single particles was initially exclusively performed on modified, home-built mass instruments (e.g., electrostatic linear ion traps,^3,4^ time-of-flight^5^) but more recently the CDMS technology was also implemented on commercial Orbitrap-based mass analyzers.^6^ Since its development around 2020, Orbitrap-based CDMS has undergone significant developments toward more accurate and sensitive charge analysis, notably by improved sample preparation and data analysis tools, and gaining access to ultralong transients.^7–9^

While Orbitrap-based CDMS performs remarkably well for high mass ions, a major challenge still lies in the detection of smaller analytes which carry fewer charges and generally fall in the lower *m*/*z* regions. These ions frequently experience numerous high-energy collisions with background ions, negatively affecting their trajectories within the Orbitrap mass analyzer.^10,11^ Slightly different approaches (e.g., stepped fast Fourier transform (FFT)^13,14^ or frequency chasing,^12^ Direct Mass Technology mode^15,16^ ) have been developed to trace ions along the whole transient and correct for conceivable frequency drifts. However, at extended transient times (>1–2 s), only a few lucky ions maintain stable ideal trajectories, as reported also on FTICR instruments.^17–19^ Under these extended trapping conditions, nonideal trajectories become the norm, and ions can deviate so far from their initial *m*/*z* that properly tracking particles becomes complicated. Prolonged trapping in the Orbitrap mass analyzer translates into a longer traveled distance, and therefore a higher probability for an ion to collide with background gas. For low *m*/*z* ions which orbit with high frequencies, a single collision with a neutral gas molecule can already result in the complete loss of the ion, as the energy transferred during the collision is high enough to cause fragmentation and expel the ions from their stable trajectory. Because of the poor survival rate of low *m*/*z* ions, it is currently impossible to take full advantage of extended transient lengths for smaller analytes. This is even more unfortunate considering that individual ions with low charges (<10+) can only be traced accurately if they benefit from the boost in signal-to-noise (*S*/*N*) ratio offered by recording ultralong transients.^7^ The detection of low molecular weight proteins may be within reach by acquiring longer transients, but improving ion stability within the Orbitrap mass analyzer is then a prerequisite. Monitoring ion decay and trajectories has revealed that lower charge states and larger ions are beneficial in surviving collisions with background gas molecules.^10–12,20^ Therefore, one avenue to increase the stability of single particles is to perform charge reduction on the analyte, which will move ions to higher *m*/*z* regions. Decreasing an ion’s frequency will reduce its orbiting path length and kinetic energy, resulting in fewer and less energetic collisions with the background gas.

The most common way of achieving charge reduction in native MS is to add charge reducing reagents in the electrospray solvent^20–22^ (typically imidazole or triethylamine acetate, TEAA). To illustrate this effect, our group recently highlighted that individual ions from empty adeno-associated virus (AAV) capsids are more likely to deviate from ideal trajectories than filled capsids, obstructing the correct determination of empty-to-filled ratios, a key-attribute in the quality control of these gene-therapy modalities.^20^ Charge reduction of the AAV ions, by adding TEAA in solution, helped to enhance the stability of individual ions in the Orbitrap and salvaged the determination of the correct empty/filled ratios, as validated in solution by mass photometry. However, the effectiveness of chemically induced charge reduction in significantly increasing *m*/*z* values remains limited, underscoring the need for alternative approaches. Several techniques enabling charge manipulation directly in the gas phase, to study intact proteins, have been reported.^23–25^ These methods can involve reactions with an electron donor or proton acceptor reagent, as is the case for electron transfer dissociation (ETD)^26^ and proton transfer charge reduction (PTCR).^27^ Alternatively, electron capture charge reduction (ECCR) can be used, relying on direct interaction of ions with free electrons to generate radical species. Until now, the charge reducing effect of ECCR has mostly benefited native MS applications, to avoid extensive peak overlap and ease the characterization of large polydisperse assemblies such as AAVs,^28^ heavily glycosylated SARS-CoV-2 spike protein trimer,^29^ or recombinant IgA1.^30^ Recently, Le Huray et al. demonstrated that ions could be pushed up to 200 000 *m*/*z* using ECCR, accomplished after minor instrumental modifications.^28^ This opens several new opportunities, notably also for CDMS, as the possibility to move ions to substantially higher *m*/*z* values should in theory translate into higher stability and survival rates of the ions.

Here, we report on the use of ECCR prior to CDMS measurements (ECCR-CDMS) to considerably improve the stability of ion trajectories in the Orbitrap analyzer. First, we describe the problem that at prolonged trapping times, smaller species from a mixture of antibody oligomers (IgG1 to (IgG1)_6_) are more prone to frequency shifts than larger multimers, affecting their mass analysis. After charge reduction by ECCR, the survival ratio of these lower molecular weight IgG1 monomer ions (∼150 kDa) increased considerably. These initial observations prompted us to perform ECCR-CDMS on even smaller analytes which generally elude conventional CDMS. Following ECCR, single particles of bovine serum albumin (BSA, ∼66 kDa, ∼8–12 charges) could be traced up to 24 s, with fewer deviations from ideal behavior in their ion trajectories, reflected by a gain of +55% in survival compared to standard charging by native MS (∼13–16 charges). Encouraged by this success, we next attempted to record individual ions of cytochrome c (∼12 kDa). Tracing those ions using our previous segmented-FT approach was almost impossible as low charges (7+) at transient duration of 1–2 s could not be reliably distinguished from the noise signals. Detecting such ions relied on recording stable trajectories for the full 24-s-long transient time. Altogether, our ECCR-CDMS coupling strongly benefits CDMS measurements by increasing ion stability and limiting deleterious drifting events, allowing to expand the range of Orbitrap-based CDMS applications to low molecular weights.

## Materials and Methods

### Sample Preparation

Both cytochrome c (from equine heart) and BSA were purchased from Sigma-Aldrich. The IgG1-RGY oligomer mixture was provided by the team of J. Schuurman (Genmab). GroEL was recombinantly expressed in-house as previously described.^31^ Samples were buffer exchanged into 150 mM ammonium acetate at pH 6.9 using Zeba spin columns (7 kDa MWCO, Thermo Fisher Scientific). Samples were further diluted in aqueous ammonium acetate prior to analysis to achieve the individual ion regime. Approximately 2 μL of each sample was loaded into in-house pulled, gold-coated borosilicate capillaries for nano-ESI.

### MS Parameters for Native and ECCR Orbitrap-Based CDMS Experiments

All MS experiments were performed on a modified Thermo Scientific Q-Exactive UHMR Orbitrap mass spectrometer equipped with an ExD TQ-160 cell (e-MSion, Corvallis, USA) placed after the quadrupole mass analyzer. The ECD cell was set to transmission-only mode for CDMS measurements performed in native conditions, i.e., without charge reduction. For ECCR-CDMS experiments, the filament current was set to 2.3 A and the ExD cell voltages corresponding to L2, LM3, L4, FB, LM5, and L6 were fine-tuned for each analyte (see Table S1).

### Data Acquisition and Processing of Ultralong Transients in Orbitrap-Based CDMS

To enable 24-s ion trapping and acquisition, an external data acquisition system FT Booster X2 (Spectroswiss, Lausanne, Switzerland) was coupled to the UHMR Orbitrap instrument, as described elsewhere.^7^ Raw transients were first processed by Peak-by-Peak software v.2023.3.1 (Spectroswiss) to generate time-domain data, which were subsequently processed with in-house Python scripts. Briefly, each 24 s-transient was divided into segments of 1 s, zero-filled four times, and apodized using a Hamming window. The processed segments were then Fourier-transformed from the time domain to the frequency domain using magnitude FT. Next, the frequency-chasing approach was used to trace single-ion signals along the 1 s segments within each 24 s-long transient.^12^ Unstable trajectories were then filtered out based on ions with a standard deviation above 1 for *m*/*z* and 0.5 for intensity. Ion survival was estimated by taking the total number of ions traced at 1 s as reference.

### Ion Stability Calculation Using Resolution as Proxy

For cytochrome c used in the present work, and more generally for low-charge ions, recording 1–2 s transient results in a very low *S*/*N* ratio. To increase the precision of centroid determination, the raw transient was processed to obtain a time-domain transient, zero-filled four times, and apodized using a Hamming window. Individual ions were detected by identifying local maxima with a specific prominence threshold determined after manual inspection of single scans. Detected signals were centroided using quadratic interpolation to refine their positions, and their full width at half-maximum (fwhm) was calculated to determine experimental individual ion resolution. To estimate a theoretical resolution (*R*) from the *m*/*z* position, we calibrated the $R=\left(f\cdot 24\cdot \sqrt{200/(m/z}\right))/1.4$ relationship to stable GroEL ions that were traced along 24 s transient, where *f* is a calibration factor. This yielded a root-mean-square deviation (RMSD) of 0.24 s. The resulting formula of $R=\left(230000\cdot 24\cdot \sqrt{200/(m/z}\right))/1.4$ , was then used to estimate theoretical *R* values for all cytochrome c ions.

## Results

### Trajectories of High Frequency Ions Do Not Endure Long Transient Recordings

To illustrate the distinct gas-phase behaviors of smaller (∼10–150 kDa) and larger particles (∼150–900 kDa), we first analyzed individual ions originating from a series of IgG1 oligomers (1 ≤ *n* ≤ 6). This mixture of species turned out to be ideal to showcase differences in the stability of trajectories of high vs low frequency ions as it covers a wide mass range from monomers (∼150 kDa) up to hexamers (∼900 kDa) (Figure 1A). Transients were recorded up to 24 s, and a frequency chasing approach was used to track each individual particle and filter out unstable ions. The resulting mass histogram shows five resolved peaks, whose masses correspond to the IgG monomer, dimer, trimer, tetramer and hexamer (Figure 1B), in line with what we published earlier.^32,33^ The three largest oligomers (*n* > 2) exhibit quite stable ion trajectories, with minimal deviations in *m*/*z* over the full 24 s transient length (Figure S1). This remarkable stability translates into what we term here as “survival ratios” > 80% for trimer, tetramer and hexamer ions, whereby only 20% of the ions do not make it to the end of the transient.

**Figure 1:**
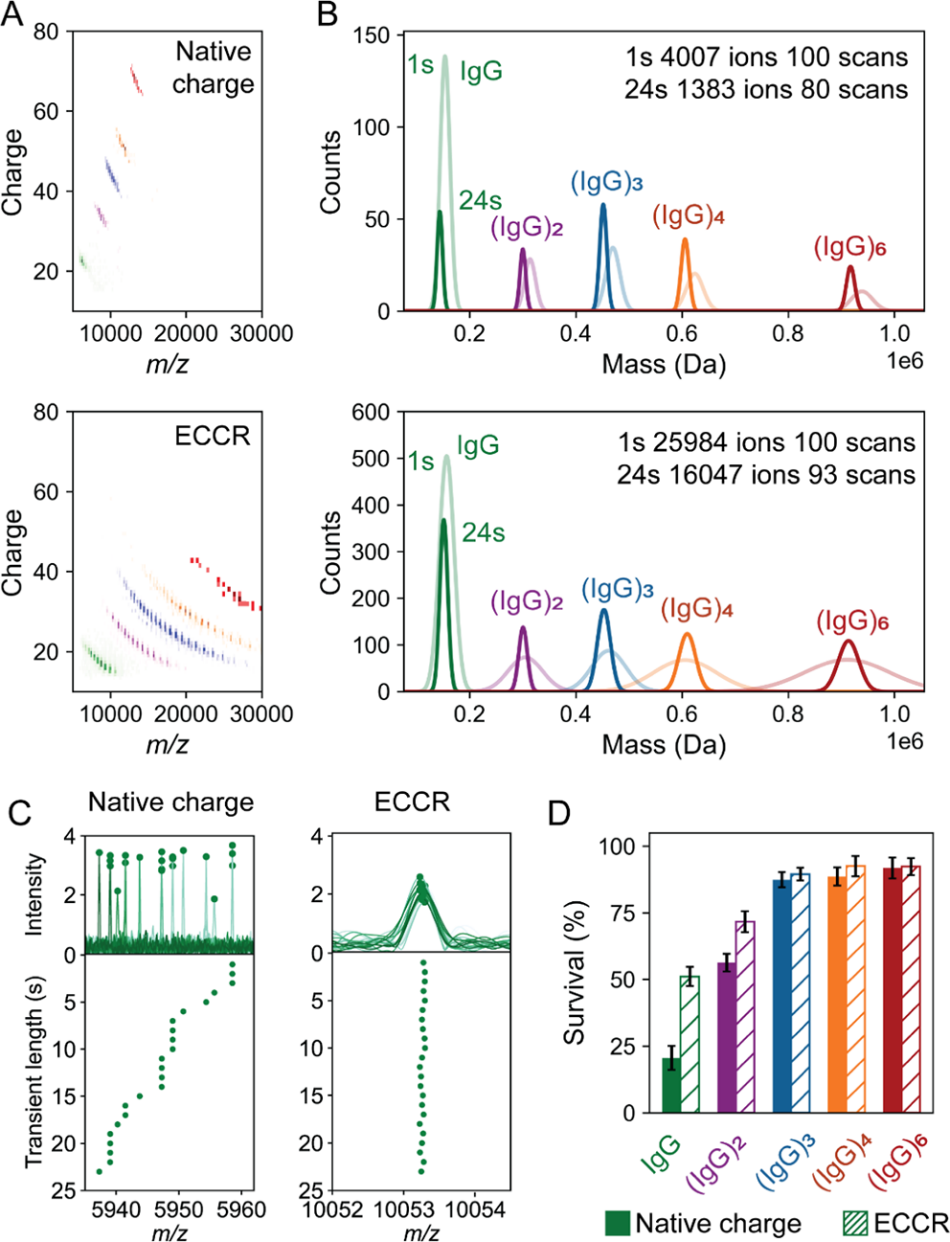
Gas phase charge reduction by ECCR enhances ion survival in Orbitrap-based CDMS. **(A)** Two-dimensional native CDMS histograms of standard charged and charge-reduced ions for IgG1 oligomers. Ion signals of different oligomeric species are color-coded in panel B. **(B)** Mass histograms derived from CDMS measurements using either 1 s (broader) or 24 s transients (narrower). Data were collected across ∼ 100 scans, and # of measured ions are depicted. **(C)** Representative stable and unstable ions of the IgG monomer. Individual ions (top) were frequency-chased by dividing the 24 s transient into 1 s segments (bottom). The native charge ion (25+, left) experienced gradual frequency (*m*/*z*) shifts primarily due to collisions with background gas that results in multiple neutral losses. After 24 s, the mass has decreased (− 500 Da), while the charge reduced (15+, right) ion displayed no shift. **(D)** Bar plot showing the ion survival (i.e., no substantial drifts) for the different IgG1 oligomers before and after ECCR. Error bars are based on technical duplicates.

In contrast, we noticed that individual ions of the IgG monomer and dimer display many drifts in *m*/*z* (Figure 1C,D). We hypothesize that any collision with background gas molecules will make them deviate from their ideal ion trajectories, as these lower molecular weight particles orbit at higher frequencies with relative higher kinetic energies and lower collision cross sections (CCS).^10–12^ A vast majority of ions indeed showed frequency drifts, even if we lowered the pressure as much as possible to minimize the number of collisions. This is reflected by low survival ratios at extended transient times, as only 20% of the IgG monomer ions were traced up to 24 s (Figure 1D). Regrettably, these nonideal behaviors in ion trajectories undermine the benefits of ultralong transient recording. Finding a way to boost ion survival, especially for small to medium sized analytes, is essential for fully leveraging prolonged transients and achieve high-resolution Orbitrap-based single molecule CDMS.

This led us to explore ECCR to improve the stability of ion trajectories over prolonged transient times. In theory, ECCR could help stabilize low-mass ions by shifting them to higher *m/z,* reducing their oscillation frequencies, velocities and, therefore, the traveled distance. Since the frequency of collisions decreases linearly with the traveled distance, shorter ion paths reduce the likelihood of collisions with gas molecules (Figure S2).^12,17^ To test this, we performed ECCR first on the IgG1 multimer mixture, giving us access to a wide distribution of charge-reduced ions for each oligomer (Figure 1A). Even with the Orbitrap analyzer’s resolution decreasing as a function of $R∼\frac{1}{\sqrt{m/z}}$ , all species were clearly baseline-resolved after 24 s, still allowing for accurate mass determination of the oligomeric series (Figure 1B). More importantly, we observed a significant gain in ion stability, increasing the survival ratio from 20% to 52% for the (150 kDa) IgG monomer (Figure 1C,D). Of note, the average charge *z* of IgG monomers is ∼25 under standard native MS conditions and reduces to ∼15 after ECCR. This first data set confirms that ECCR may provide a powerful approach to enhance the stability of the ion trajectories of higher frequency ions without impairing the charge accuracy (Figure S3A,B), suggesting that ECCR could facilitate the analysis of even smaller analytes (<100 kDa) that are normally out of the reach for standard Orbitrap-based CDMS.

### ECCR Improves Dramatically the Stability of Ion Trajectories of Smaller Analytes

Our initial results with the IgG mixture encouraged us to analyze lower molecular weight proteins, starting with bovine serum albumin (BSA (∼66 kDa). In our earlier work where we introduced ultralong transients,^7^ we already reported that under native conditions, only a small fraction of BSA ions survived the full 24 s transient. This was attributed to their high oscillation frequencies, which posed significant challenges as described above. ECCR in principle offers a way to improve the survival rate of smaller proteins like BSA, potentially addressing the limitations highlighted in our earlier study.

To investigate this, we recorded individual ions of BSA under native (Figure 2A) and charge-reduced (Figure 2B) conditions at identical pressure settings. We then calculated the ion survival under both conditions over the 24 s-long transients. This analysis first confirms that regardless of the ion charge, the survival rate decreases over the transient as the ions experience more and more collisions with background gas molecules (Figure 2C). Nevertheless, the drop in ion survival is significantly greater for natively charged ions compared to charge-reduced ions. Only 25–50% of the native BSA ions (16 ≤ *z* ≤ 13) survive the entire 24 s transient, whereas the survival rate for ECCR BSA ions (8 ≤ *z* ≤ 12) increases to as much as 80% (Figure 2C).

**Figure 2:**
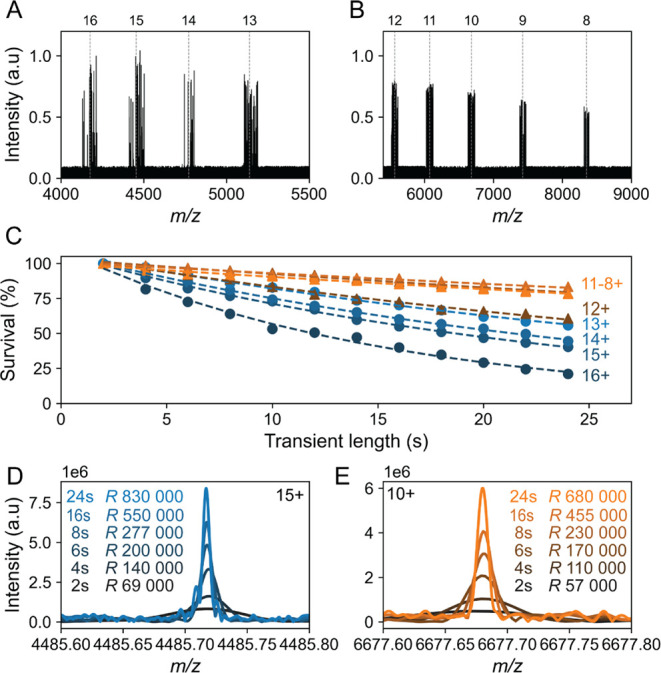
CDMS measurements for BSA at extended transient times (24 s). Individual ions of BSA **(A)** recorded in their native charge state and **(B)** charge-reduced after ECCR. **(C)** Survival rate of ion trajectories of individual BSA ions along the 24 s transient. The frequency-chasing approach was used to monitor ion stability along the trajectory, defining stable ions as those with a standard deviation <1 *m*/*z* over the transient length. Comparing the number of stable ions between the first and subsequent segments provides an estimation of ion survival. **(D)** Signal of a 15+ and **(E)** 10+ BSA individual ions at increasing transient lengths.

However, it is important to acknowledge that reducing charge states comes with a trade-off, as the Orbitrap resolving power is reduced when ions are translated to higher *m*/*z* regions. For instance, a stable native 15+ ion shows a linear increase in mass resolution with transient length, reaching *R* ∼830 000 (Figure 2D). For a charge-reduced 10+ ion, the maximum achievable *R* is ∼680 000. Although this represents an 18% reduction in *R* compared to the 15+ ion, it remains theoretically possible to resolve mass differences smaller than 1 ppm (Figure 2E). Altogether, the benefits brought by performing ECCR-CDMS at extended transient times clearly surpass the concomitant loss in resolving power.

### Closing in into the Detection of Singly Charged Ions Using Orbitrap-Based CDMS?

Under standard conditions only a handful of individual ions from cytochrome c (∼12 kDa) survive the first seconds of a transient in the Orbitrap mass analyzer (8 s at best).^7^ Using ECCR-CDMS, we next aimed to improve the detection of such analytes as well. If also charge-reduced cytochrome c ions would attain more stable ion trajectories, we should possibly be able to tackle the detection of ions with *z* < 5, getting closer to measuring ultimately individual singly charged ions by pushing the limits of Orbitrap-based CDMS, a feat only shown before by using an electrostatic linear ion trap (ELIT) designed for CDMS.^34^

Detecting and tracing ions with charge *z* < 10 through frequency chasing proved to be too challenging. Such an approach relies on dissecting a transient in e.g., segments of 1 s, which for such low charged ion yields a *S*/*N* < 3, very close to the detection limit (*S*/*N* ∼3 after 1 s for a *z* = 7 ion on the Orbitrap UHMR instrument, Figure 3A). This means that noise peaks can falsely become traced instead of true ion signals, introducing significant uncertainty in signal detection. To address this challenge, we did not perform a segmented-FT analysis but instead processed only the full-length transients (24 s). As the *S*/*N* scales with the square root of the transient length, the *S*/*N* improves ∼5-fold moving from 1 to 24 s transients, enabling more accurate centroid detection, diminishing erroneously picked ion signals (Figure 3A).

**Figure 3:**
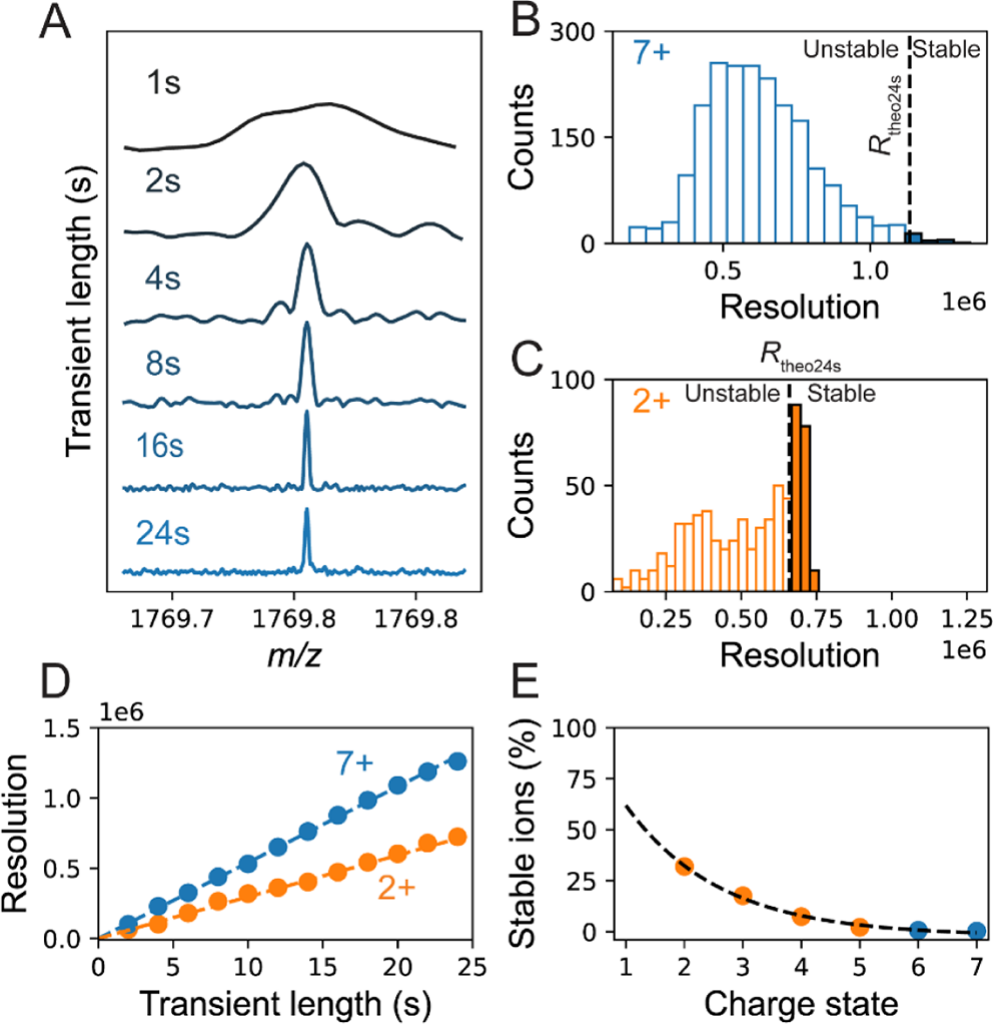
Measured resolution as proxy to estimate the stability of ions. **(A)** CDMS data of *z* = 7 individual ions of cytochrome c (∼12 kDa) at different transient lengths. At short transients (1 and 2 s), the *S*/*N* of the ion is too low for accurate ion tracing, which can be resolved by processing instead the full 24 s. Using a full FT approach, histograms of the experimentally determined resolutions for each of the individual ions can be constructed. The theoretical resolution is given as a vertical dashed black line. In **(B)** the histogram is depicted for *z* = 7 ions of cytochrome c and shows that most ions do not reach the theoretical limit. **(C)** Alike (B), but now for the charge-reduced *z* = 2 ions of cytochrome c, again at 24 s transients. The empty and fully colored bins represent ions with unstable and stable trajectories, respectively. A “stable ion” was defined by comparing the experimental resolution to the theoretical resolution (indicated by the dashed line) (see also Figure S4). **(D)** Resolution of cytochrome c *z* = 7 (blue) and *z* = 2 (orange) ions, at increasing transient lengths. The experimental data follow the theoretical resolution (dashed line) calculated using the established calibration curve $R=\left(230000\cdot 24\cdot \sqrt{200/(m/z}\right))/1.4$ . **(E)** Relationship between the charge state of cytochrome c ions and their stability. The blue and orange points represent charge states formed for cytochrome c under standard native and ECCR conditions, respectively. Data were fitted to an exponential function (*R* ^2^ > 0.98).

To assess the stability of individual ions, we used the experimentally determined resolution for each individual ion signal as a proxy for ion survival. For ions having stable ion trajectories, the resolution should increase linearly with transient length. Reversely, if the ion does deviate substantially from the ideal trajectory over the selected transient time, the experimental resolution will be lower than the theoretical resolution. First, to estimate the theoretical resolutions at a given transient time, we measured and used a reference data set, relying on highly stable GroEL ions to calibrate the *R* ∼*m*/*z* ^–1/2^ dependency (see **Materials and Methods**, Figures S4–S6). Following this calibration, we were able to predict precisely the theoretical resolution for each *m*/*z* of cytochrome c at a selected 24 s transient time, filter stable ions and built mass histograms (Figure S7). Under standard native conditions, the most abundant charge state observed for cytochrome c is *z* = 7. After recording CDMS measurements for 2h, fewer than 1% of the detected ions (10 ions) achieved the expected *R* for charge state *z* = 7 (Figure 3B). On average, ions exhibited a *R* of ∼600 000, which translates to ions traveling stable trajectories on average for ∼11 s in the Orbitrap. For *z* = 6, only 2 ions displayed the expected theoretical *R*. Even by combining ultralong transients and our full FT approach to enhance the *S*/*N* and improve charge accuracy (Figure S3), the intrinsically low stability of the ion trajectories of cytochrome c ions with *z* = 6 or 7 remained a limiting factor to measure them by CDMS.

Applying ECCR we were able to generate abundant *z* = 2 ions for cytochrome c. Recording such signals improved the ion statistics substantially by a factor of 60, resulting after 2 h in a total of ∼600 stable ions across all detected charge states, among which ∼100 corresponded to *z* = 2, with a *S*/*N* of ∼12 after 24 s (Figure 3C). For such low charges, improving the *S*/*N* especially benefits the accuracy of charge assignment, and alike values are obtained under native (σ_7+_ = 0.5 charges) and charge reduced (σ_2+_ = 0.4 charges) conditions (Figure S3C). Of note, although stable *z* = 7 ions can achieve a *R* ∼ 1 000 000 at 24 s, *z* = 2 ions can still reach nearly a *R* ∼730 000 at 24 s, enabling to resolve differences of 0.00822 *m*/*z* (Figure 3D). By inspecting the behavior for ions of each charge state, we established a relationship between ion trajectory stability and charge state, with ion survival increasing 100-fold from 0.3 to 32% as *z* diminished from 7 to 2 (Figure 3E).

### Understanding the Distinctive Factors Influencing Single-Ion Stability in Orbitrap

To better understand the differences in ion survival across charge states and analytes, we quantified the number of collisions as a function of *m*/*z* or frequency (Figure 4A). At constant pressure, collision probability depends on the traveled distance and CCS. For globular proteins such as BSA under native MS, CCS exhibits minimal variation across charge states, implying that the collision probability is predominantly influenced by the traveled distance. As shown in Figure S2A, lower charge states for a given analyte oscillate at lower frequencies and thus travel shorter distances, thereby undergoing fewer collisions. To exemplify, after 24 s, native 15+ BSA ions (*m*/*z* ∼4500) traveled ∼277 km experiencing ∼10 collisions, while a charge-reduced 10+ BSA ion (*m*/*z* ∼7500) traveled ∼214 km and underwent ∼8 collisions (Figure 4A). Despite only two fewer collisions, the 10+ ions exhibited significantly greater stability (80% vs 50%) (Figure 2C). This *z*-dependent survival differences are further explained by variations in ion kinetic energy, determined by the product of the ion’s charge and the potential difference between the C-trap and the Orbitrap outer electrode.^17^ Because charge-reduced ions have lower kinetic energy, they experience both fewer collisions, and lower center-of-mass (COM) energy transfer during collisions with background gas molecules. In this example, 15+ BSA ions absorb 3.2 V, while 10+ ions absorb 2.1 V (Figure 4B). This combined reduction is even more pronounced for cytochrome c ions, where the energy transferred in a single collision with a low *m*/*z* ion is high enough to cause ion decay. Charge reduction dramatically decreases the energy transfer per collision, enabling these ions to survive.

**Figure 4:**
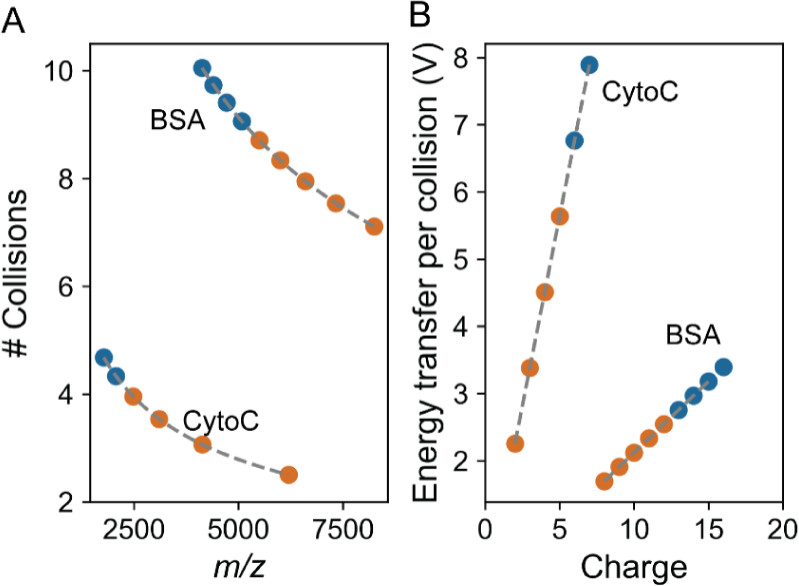
Factors affecting ion survival. Number of collisions as a function of the *m*/*z* **(A)** and the energy transfer per collision as a function of the charge **(B)** for BSA and cytochrome c ions. Low mass ions experience fewer collisions but undergo higher energy transfer per collision, impairing ion survival. The average number of collisions and energy transfer per collision are calculated as previously described^12^ using a pressure of 1.58 × 10^–11^ mbar and a radii of 2.2 and 3.7 nm for cytochrome and BSA, respectively. The blue and orange points represent charge states formed under standard native and ECCR conditions, respectively.

## Discussion and Conclusions

Here we explored novel means to improve the trajectories of individual ions within the Orbitrap mass analyzer with the aim to improve key aspects of Orbitrap-based CDMS. More stable ion trajectories allow to record the image currents for longer times, which directly enhance the *S*/*N* and thus the sensitivity. Boosting the *S*/*N* not only enhances the accuracy of charge state determination but also helps overcome a key limitation of Orbitrap-based CDMS. Because the signal scales with ion charge, analyzing ions that carry just a few charges has been notably difficult, hampering the application of CDMS to smaller proteins and peptides. These smaller proteins and peptides exhibit typically lower *m*/*z* values than protein assemblies generated by native MS, which directly relates to their higher orbiting frequency, and thus velocity. Moreover, with longer transient recordings, ions are more likely to collide with the residual gas molecules in the Orbitrap. Each of these collisions can have a detrimental effect on the stability of the ion trajectory. All these features have led to the fact that Orbitrap-based CDMS has been very successful for the mass analysis of macromolecular complexes such as IgM,^35,36^ AAVs,^37,38^ GroEL and ferritin,^7^ but has had less of an impact in the analysis of medium sized and small proteins, let alone peptides. Broadening the applicability of Orbitrap-based CDMS would facilitate the analysis, tentatively at single molecule sensitivity, of complex, low-abundance samples that may contain both small and large molecules.

Previously, both others and we reported that high mass ions have generally more stable ion trajectories in the Orbitrap, and subsequently also observed that this stability seemingly increased when these ions were moved to higher *m*/*z* (i.e., lower charge).^7,12,17,21^ These observations led us to here explore a relatively novel approach, termed electron capture charge reduction (ECCR), to charge reduce ions in the gas phase. Just recently the groups of Wysocki^29^ and Sobott^28^ demonstrated that by using ECCR, ions of large macromolecular complexes can be efficiently moved to very high *m*/*z* values (up to 200 000), which also helps in deconvolving heterogeneous signals in native MS, such as those for highly glycosylated viral Spike proteins^29^ and IgA.^30^ Indeed, also in the present work, we observed that ECCR can be used to efficiently charge reduce ions, as next to electron-capture induced fragmentation, most precursor ions capture multiple electrons without dissociation, sometimes called ECnoD.^39–41^ In addition, ECCR predominantly neutralizes exposed protons in a charged gas-phase protein, reducing the additional energy of Coulomb explosion, and therefore decreasing the probability of fragmentation/breaking off of outer chains upon collision with residual gas.

Evidently there are alternative ways to produce ions with lower charge states either at the initial stage of ionization or in the gas phase. First, MALDI naturally generates primarily singly charged ions (accompanied sometimes by doubly and triply charged ions), but this may be overarching for current possibilities in CDMS. In native MS, many additives have been added to the aqueous ammonium acetate solution with the aim to charge reduce the ions further during the ionization process. An issue with this chemically induced charge reduction is that many of these chemicals seem to stick to the produced macromolecular ions, which in CDMS may lead to them becoming desolvated during the extended trapping in the Orbitrap, affecting their stable ion trajectories as described here. Finally, as mentioned before, PTCR may provide a way to charge reduce ions in the gas phase, similar to ECCR. Potentially, it may even outperform ECCR as the proton transfer is less energetic than an electron capture, and thus in theory PTCR should induce less fragmentation than ECCR. There may therefore still be room for improvement.

Recording longer transients in FTMS allows to improve both the resolution and sensitivity in Orbitrap-based CDMS, as we recently demonstrated.^7^ Prolonged trapping should allow for the detection of lower charged smaller ions but in practice, low *m*/*z* ions tend to easily lose their coherent motion in the Orbitrap. As we demonstrate here clearly, such unstable trajectories, caused by ion decay, frequency drifts, or collision-induced fragmentations limit the benefits of recording longer transient times. Here, we report the implementation of ECCR prior to Orbitrap-based CDMS and demonstrate that this can improve ion stability, enabling the trapping and detection of even doubly charged ions with astonishing resolution (730 000). But can we also reach the ultimate *z* = 1 limit in Orbitrap-based CDMS, as now only demonstrated in ELIT-based CDMS?^34^

Speculatively, by extrapolating the fit shown in Figure 3E, we estimate that around 60% of singly charged cytochrome c ions (at ∼12 000 *m*/*z*) could survive the entirety of a 24 s-long transient in the Orbitrap UHMR. Of course, this is only achievable under ultrahigh vacuum conditions. In addition, on the Orbitrap UHMR, a *z* = 1 ion would barely appear above the noise (*S*/*N* ∼2) even after 24 s, meaning such ions would only be traceable using a full FT approach, requiring still delicate manual inspection of the full data set to avoid mistakenly picking signals that are noise. Therefore, although detecting single charged ions is within reach for Orbitrap-based CDMS this can only be achieved at even higher vacuum conditions and would benefit from recording even longer transients. Unfortunately, implementing either option is not yet experimentally straightforward.

## Supplementary Material



## Notes

MS parameters used for ECCR-CDMS measurements on the Q-Exactive UHMR Orbitrap; A representative; several frequency-chased single-ion mass spectra for different IgG1-RGY oligomers; calculations of the effect of charge reduction on the estimated distance traveled and number of collisions encountered; a scheme of the strategy employed to use the extracted mass resolution as a proxy for ion survival; calibration of single-ion resolution using GroEL ions generated by standard native MS and ECCR; plots depicting stable vs unstable ion trajectories of individual GroEL ions; two-dimensional *m*/*z* vs charge and mass histograms of single cytochrome c ions recorded under standard charge and charge-reduced conditions; histograms evaluating the precision in charge determination (PDF)
