# Supplementary material for: Electron Capture-Induced Charge Reduction Benefits the Recording of Ultralong Transients in Orbitrap-Based Individual-Ion Mass Spectrometry
Source: Anal Chem. 2025 May 29;97(22):11653–60. doi: 10.1021/acs.analchem.5c01000 (PMC12163872; doi:10.1021/acs.analchem.5c01000)
Supplement: Supplementary file 1 [file ac5c01000_si_001.pdf]

Supporting Information

Electron capture induced charge reduction benefits the recording of ultralong transients in Orbitrap-based individual-ion mass spectrometry

Manuel D. Peris-Díaz [a,b], Arjan Barendregt[a], Tobias P. Wörner[c], Kyle L. Fort[a,c], Alexander A. Makarov[a,c], Evolène Deslignière[a]*, Albert J. R. Heck [a]*

[a] Biomolecular Mass Spectrometry and Proteomics, Bijvoet Center for Biomolecular Research and Utrecht Institute for Pharmaceutical Sciences, Utrecht University, Utrecht 3584 CH, The Netherlands.

[b] Department of Chemical Biology, Faculty of Biotechnology, University of Wrocław, Wrocław 50-383, Poland.

[c] Thermo Fisher Scientific GmbH, Hanna-Kunath-Straße 11, 28199 Bremen, Germany.

The supporting information contains 1 Supplementary Table S1 and 5 Supplementary Figures S1 to S7.

Table S1. MS parameters used for ECCR-CDMS measurements on the Q-Exactive™ UHMR Orbitrap™ mass spectrometer.

**MS parameters**	**CDMS**			**ECCR-CDMS**		
Sample	IgG	BSA	CytoC	IgG	BSA	CytoC
Capillary voltage (kV)	1.5	1.6	1.5	1.5	1.4	1.5
m/z range	5000-20000	3000-12000	1000-4000	7500-30000	3000-12000	1500-6000
Ion injection time (ms)	1	1	1	2	5	20
Microscans	1	1	1	1	1	1
S-Lens RF	200	200	50	200	200	50
Averaging	0					
Noise threshold						
In-source CID	20	0	0	20	0	0
In-source trapping voltage (V)	-75	0	0	-75	0	0
HCD voltage (V)	50	70	5	50	70	5
Trap gas setting	2	0.2	0.1	2	0.2	0.1
UHV readout (mbar)	1.32×10^{-10}	1.58×10^{-11}	1.54×10^{-11}	1.46×10^{-10}	1.43×10^{-11}	1.46×10^{-11}
Collision gas	N_2					
Injection flatapole (V)	4					
Inter-flatapole (V)	2					
Bent flatapole (V)	2					
Ion transfer target	High *m/z*		Low *m/z*	High *m/z*		Low *m/z*
Detector optimization			High *m/z*			High *m/z*
L1 (V)	0.2			0	0	
L2 (V)	2.8			-25	-44	
LM3 (V)	-11			8	8	
L4 (V)	2.2			9	9	
FB (V)	-2.3			1	1	
LM5 (V)	-9.4			8	8	
L6 (V)	1.5			-25	-44	
L7 (V)	-0.4			0	0	

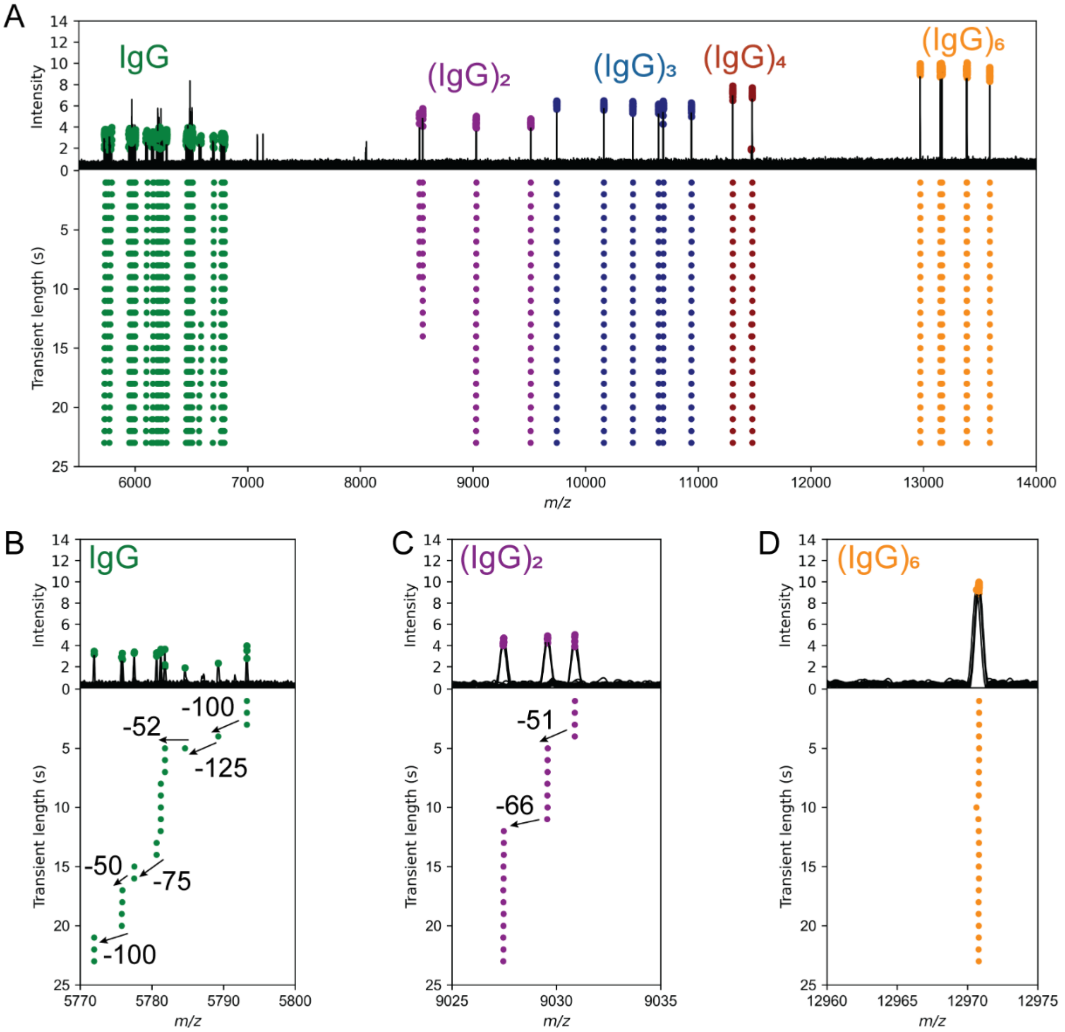

Figure S1. Monitoring ion trajectories for ions generated by standard native ESI for different IgG1-RGY oligomers. (A) A representative CDMS spectrum showing frequency-chased individual ions, color-coded per oligomer they represent. Several frequency-chased individual ion mass spectra display gradual neutral losses due to collisions with background gas for ions from **(B)** the IgG monomer (green), and **(C)** IgG dimer (purple). Conversely, single ions for the trimer, tetramer, and hexamer exhibit relatively stable ion trajectories (**A** and **D**).

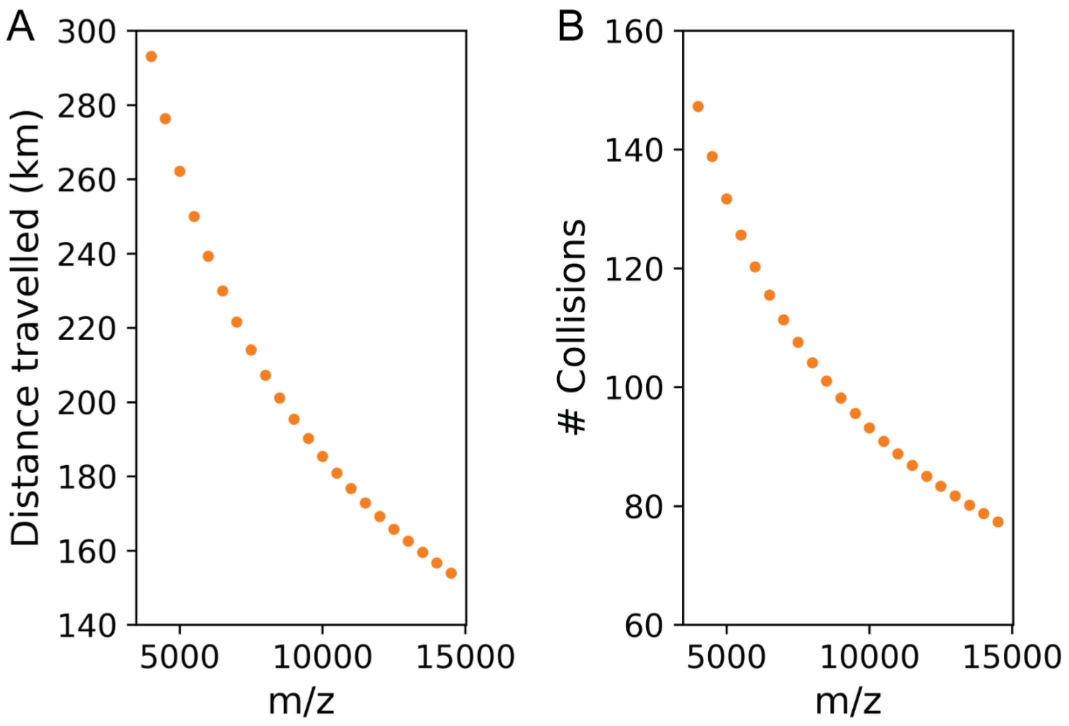

Figure S2. Effect of charge reduction on the estimated distance travelled and number of collisions encountered. The distance travelled **(A)** and the average number of collisions with nitrogen gas molecules **(B)** of monomeric IgG ions, trapped in the Orbitrap mass analyzer for 24 s, are shown as a function of *m/z*. The calculations were performed as described in (Wörner et al., 2022). The radii used for IgG was 5 nm, and the ultra-high-vacuum readout was 1.46×10^{-10} mbar.

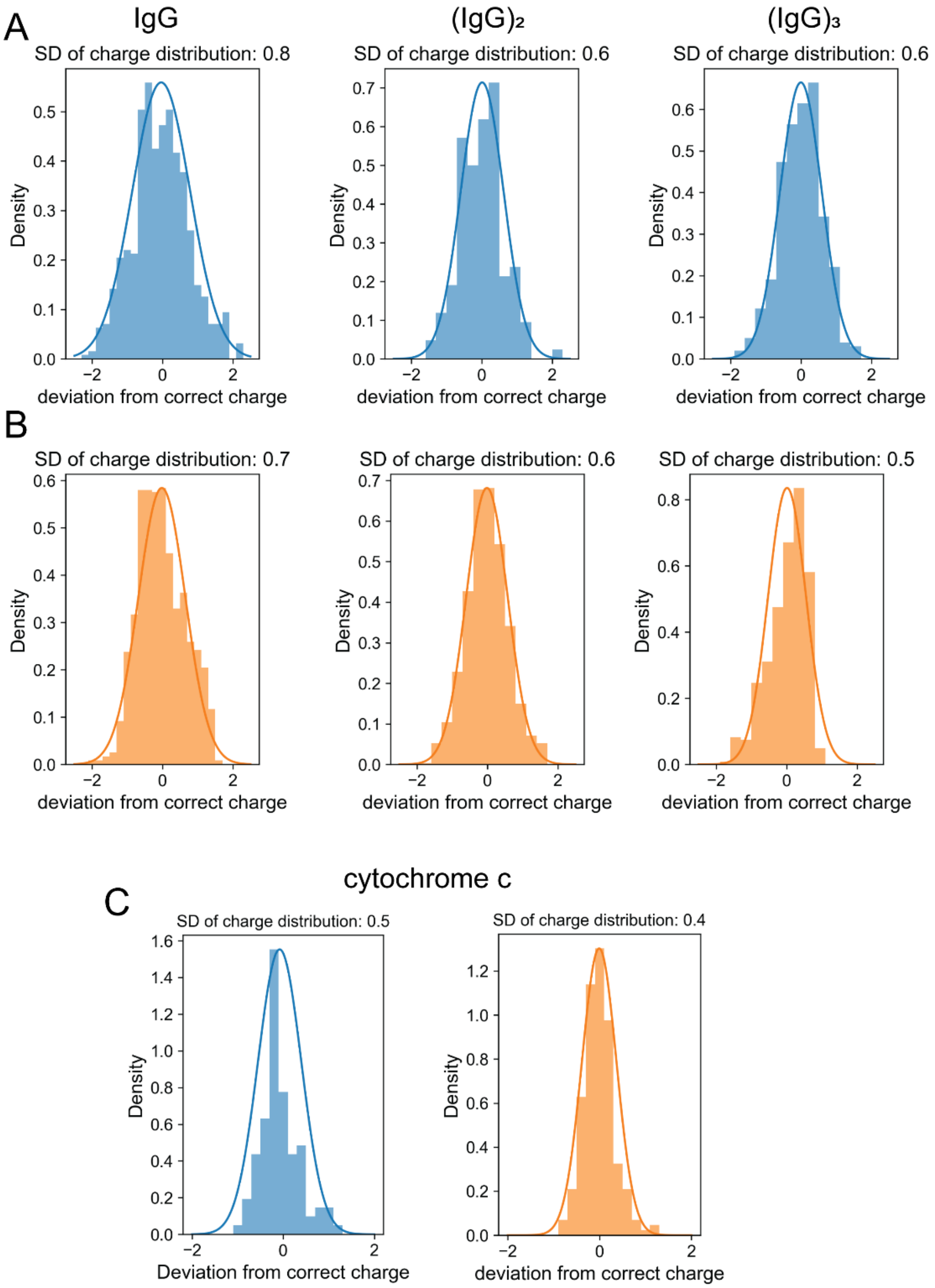

Figure S3. Precision in charge determination after 24 s. Histograms of the average charge error, defined as the difference between expected and experimental charge, for single ions originating from the IgG monomer, dimer and trimer. Data for the IgG oligomers sprayed under standard conditions are depicted in (**A**) and for charge-reduced conditions in (**B**). (**C**) Alike histograms for cytochrome c 7+ (left, standard) and 2+ (right, charge-reduced).

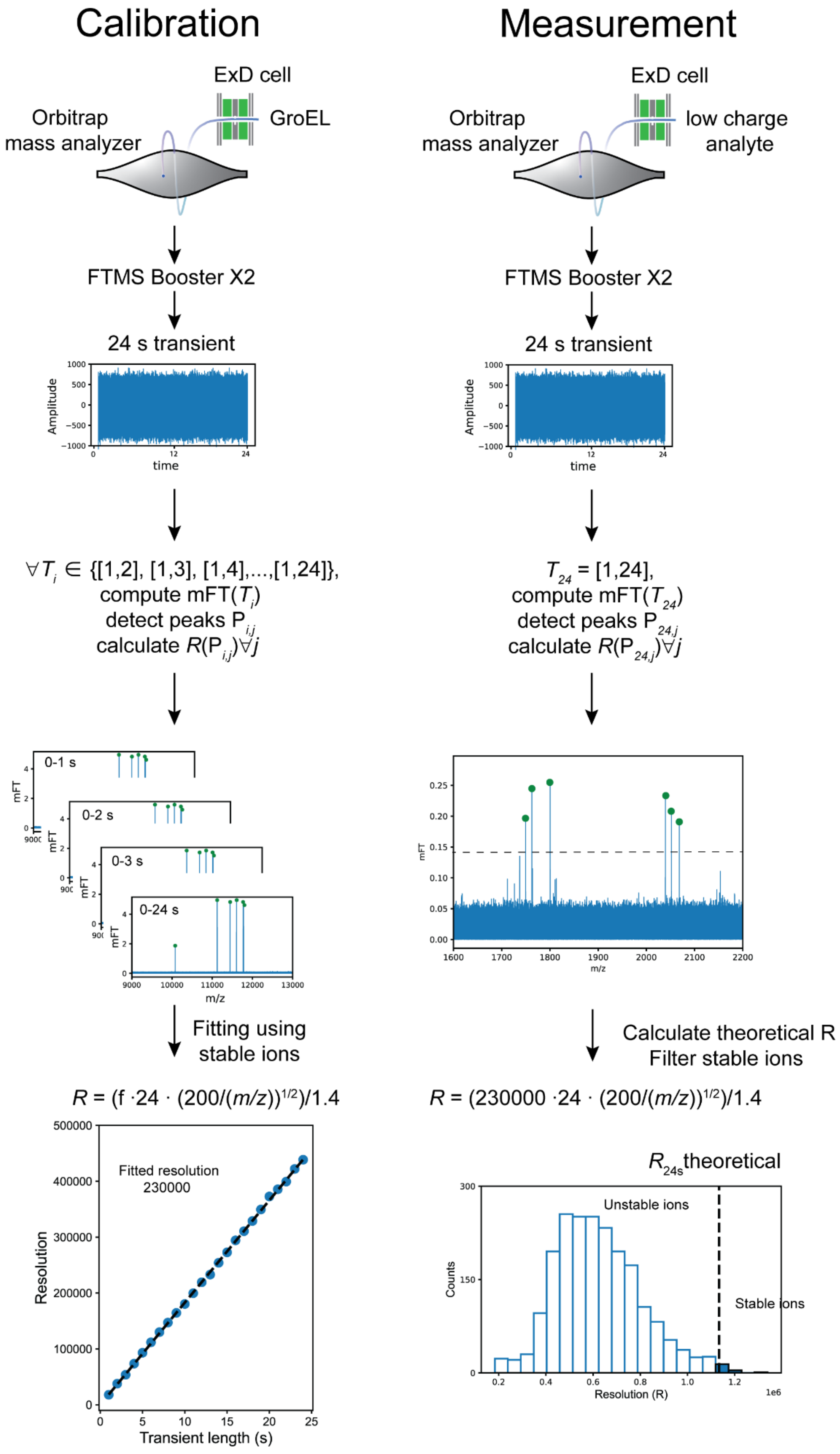

Calibration
Measurement
ExD cell
Orbitrap mass analyzer
GroEL
low charge analyte
FTMS Booster X2
24 s transient
Amplitude
time
∀T_i ∈ {[1,2], [1,3], [1,4],...,[1,24]}, compute mFT(T_i) detect peaks $P_{i,j}$ calculate $R(P_{i,j})$∀j
T_{24} = [1,24], compute mFT(T_{24}) detect peaks $P_{24,j}$ calculate $R(P_{24,j})$∀j
0-1 s
0-2 s
0-3 s
0-24 s
mFT
m/z
Fitting using stable ions
Calculate theoretical R Filter stable ions
$R = (f \cdot 24 \cdot (200/(m/z))^{1/2})/1.4$
$R = (230000 \cdot 24 \cdot (200/(m/z))^{1/2})/1.4$
Fitted resolution 230000
Resolution
Transient length (s)
R_{24s} theoretical
Unstable ions
Stable ions
Counts
Resolution (R)
1e6

Figure S4. Strategy employed to use the extracted mass resolution as a proxy for ion survival. To calibrate the resolution as a function of the *m/z* position, 24 s transients were recorded for individual GroEL ions of a variety of charge states as generated by standard native MS with and without ECCR. Cumulative segments of 1 s each were extracted from the 24 s transients, zero-filled four times, and apodized using a Hamming window. Then, the magnitude Fourier Transform (mFT) was computed to transform the time-domain signal to the frequency domain, and individual ions were frequency-chased along the 1 s segments. The resolution (*R*) defined as (*m/z*)/FWHM was determined for each centroid, and plotted against the transient length. Least squares regression was used to fit the *R* against the transient length using the $R_t = (f \cdot t \cdot (200/(m/z))^{1/2})/ 1.4$ formula, where *t* is the transient length. Alternatively, the *m/z* position and resolution of the centroids were plotted and fitted against the $R_t= (f \cdot t \cdot (200/(m/z))^{1/2})/ 1.4$ formula, determining the calibration factor *f* (**Figure S5**). This yielded a root mean square deviation (RMSD) of 0.24 s. Several examples of ions with stable and unstable trajectories are shown in **Figure S6**. To determine the survival of low-charge ions, the acquired 24 s transients were processed using mFT, zero-filled four times, and apodized. Single ions were detected, and their resolution was estimated. The formula $R_{24}= (230000 \cdot 24 \cdot (200/(m/z))^{1/2})/1.4$ was used to estimate the theoretical resolution for a 24 s transient (R_{24}). An ion was defined as stable if its R_{24} was greater than or equal to the theoretical $R_{24}- R_{0.24}$.

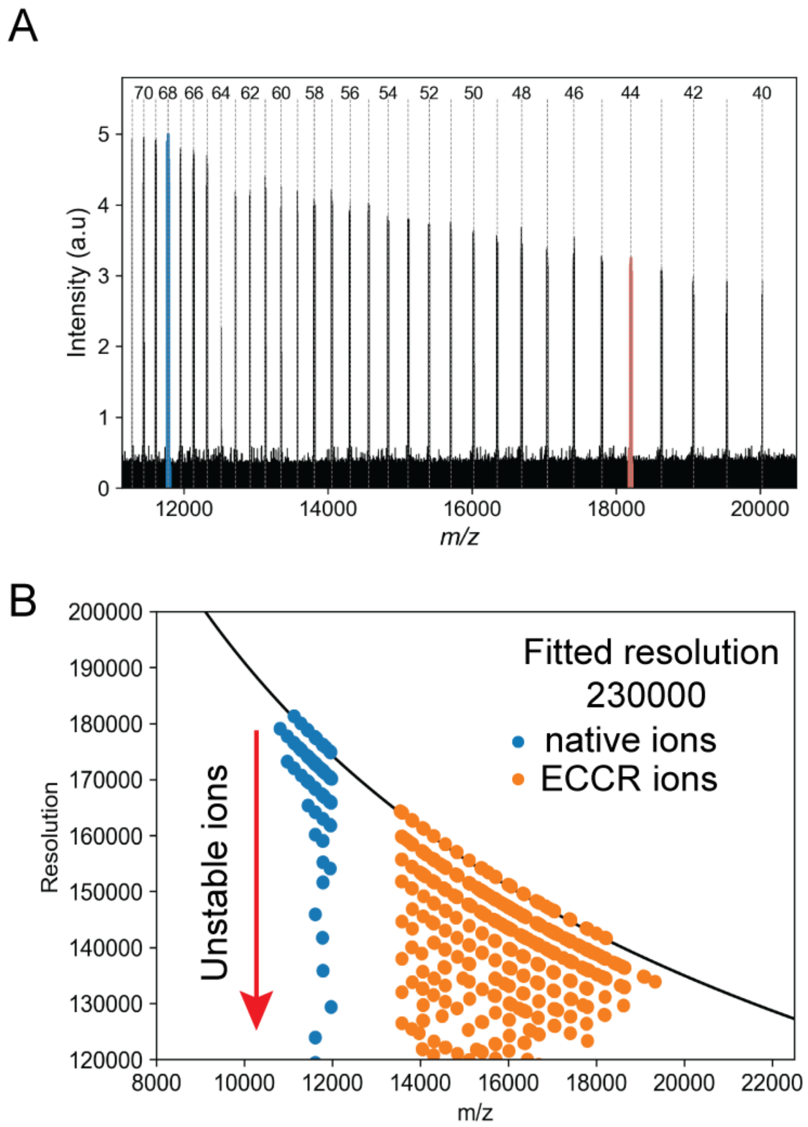

Figure S5. Calibration of single-ion resolution. (A) Composite CDMS spectrum of GroEL ions generated by standard native MS before ($71 \leq z \leq 64$) and after ECCR ($63 \leq z \leq 40$). **(B)** Estimated single-ion resolution extracted from all scans for a 24 s transient. The theoretical resolution was estimated by fitting the R vs. m/z relationship using the formula $R_t = (f \cdot t \cdot (200/(m/z))^{1/2})/1.4$. The plot depicts also several individual ions that did not survive the full 24 s and displayed lower resolution than theoretically expected. This is shown in greater detail in **Figure S6**.

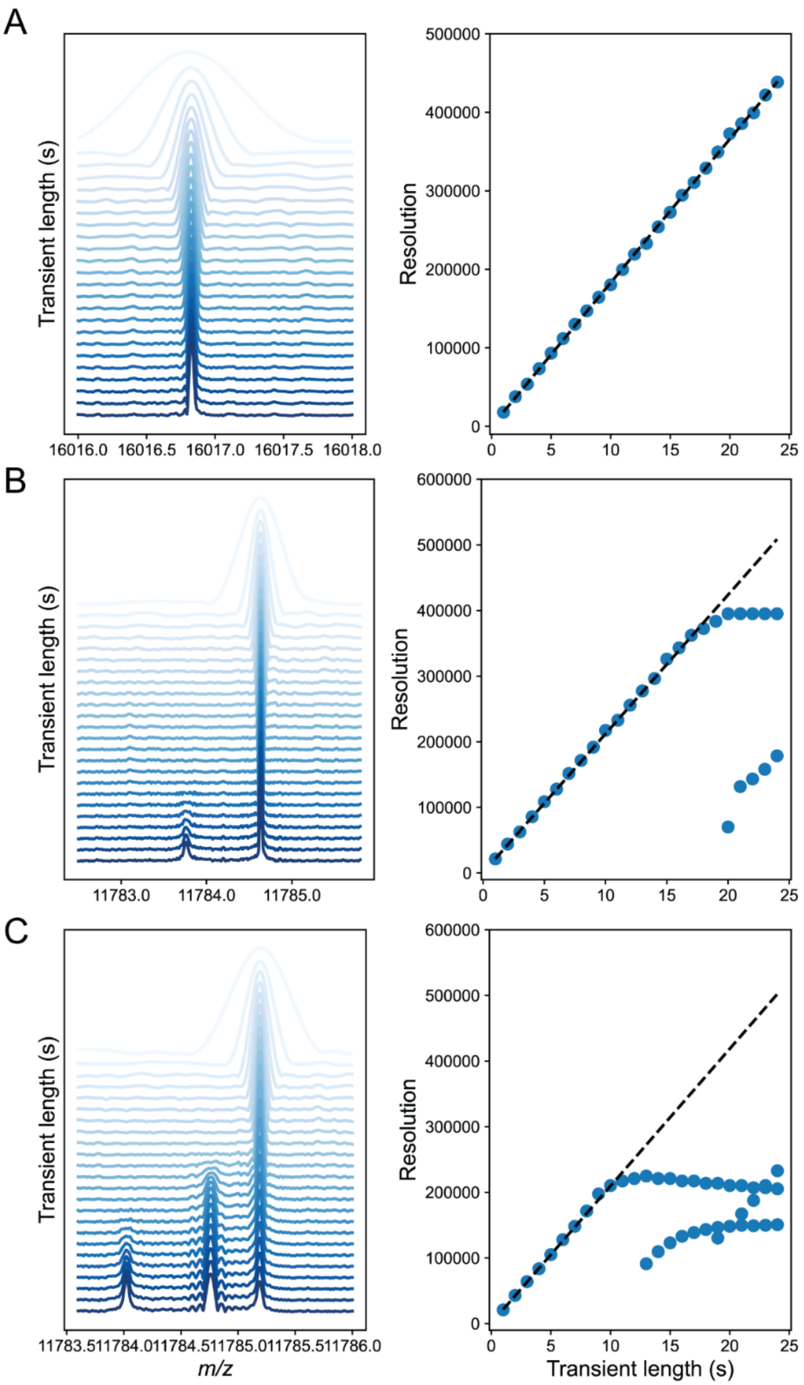

Figure S6. Stable *vs* unstable ion trajectories of individual GroEL ions. (A) The resolution of stable ions that do not experience frequency drifts scales linearly with the transient length. Unstable trajectories **(B, C)** caused by ion decay, frequency drifts, or collision-induced fragmentation result in a resolution after 24 s transient length that is lower than theoretically expected. In **(B)**, the resolution of the 11784.7 *m/z* ion stops increasing linearly after 20 s, while the 11785.25 *m/z* ion **(C)** survived only for 10 s.

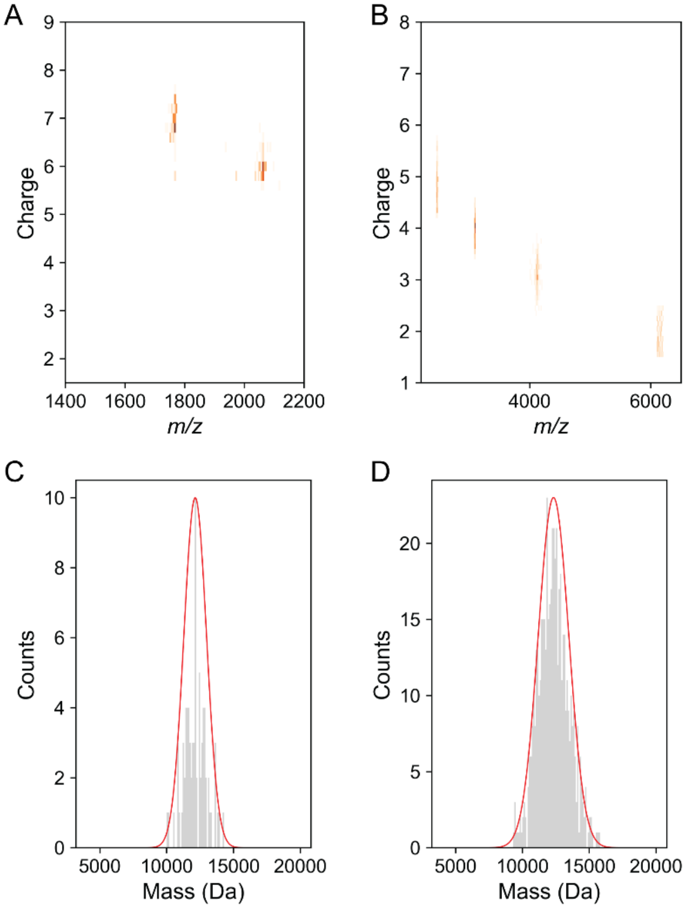

Figure S7. Single-ion CDMS of cytochrome c. Two-dimensional *m/z* vs. charge histograms and mass histograms of single ion signals recorded under standard charge (**A** and **C**) and charge-reduced (**B** and **D**) conditions.

References

1. Wörner, T. P.; Aizikov, K.; Snijder, J.; Fort, K. L.; Makarov, A. A.; Heck, A. J. R. Frequency Chasing of Individual Megadalton Ions in an Orbitrap Analyser Improves Precision of Analysis in Single-Molecule Mass Spectrometry. *Nat. Chem.* **2022**, *14*, 515–522.
